# 环糊精超交联聚合物纤维顶空固相微萃取-气相色谱-质谱法分析植物油中的邻苯二甲酸酯

**DOI:** 10.3724/SP.J.1123.2024.01019

**Published:** 2024-09-08

**Authors:** Pengcheng ZHANG, Yuan WANG, Kunling LIU, Yaming SUN, Lijun HE, Wenjie ZHAO

**Affiliations:** 1.河南工业大学化学化工学院,河南郑州 450001; 1. School of Chemistry and Chemical Engineering, Henan University of Technology, Zhengzhou 450001, China; 2.宁夏计量质量检验检测科学研究院,宁夏银川 750411; 2. Ningxia Academy of Metrology and Quality Inspection, Yinchuan 750411, China

**Keywords:** 环糊精超交联聚合物, 顶空固相微萃取, 气相色谱-质谱, 邻苯二甲酸酯, 植物油, cyclodextrin-based hypercrosslinked polymers, headspace solid-phase microextraction (HS-SPME), gas chromatography-mass spectrometry (GC-MS), phthalate esters, vegetable oils

## Abstract

邻苯二甲酸酯(PAEs)因具有改善塑料柔软性和柔韧性的特性而被作为增塑剂广泛应用于各种食品包装材料,然而,其对人类健康的重大危害使得建立准确的分析方法显得至关重要。为了解决油性基质样品前处理费时费力的问题,我们开发了一种基于顶空固相微萃取(HS-SPME)结合气相色谱-质谱(GC-MS)检测的分析方法,用于快速检测植物油基质中的12种PAEs。将苄基化环糊精超交联聚合物(BnCD-HCP)涂敷在不锈钢纤维上制备了一种具有较大比表面积、良好稳定性好和较高重复性的固相微萃取探针,并对其结构、形貌进行了表征。将油脂样品以甲醇改性后以该探针顶空萃取PAEs,优化得到如下最佳萃取参数:萃取时间20 min,萃取温度50 ℃,解吸时间4 min,解吸温度275 ℃。在优化的分析条件下,12种目标PAEs在线性范围内具有良好的线性关系(相关系数(*R*^2^)均大于0.99),检出限和定量限分别为0.21~3.74 μg/kg和0.69~12.34 μg/kg,单针的相对标准偏差(RSD)≤11.4%,针间RSD≤13.9%。将此方法应用于大豆油、花生油和葵花油样品中PAEs的检测,加标试验结果表明该方法具有良好的精密度(RSD为1.17%~11.73)和回收率(72.49%~124.43%)。该研究建立的基于BnCD-HCP的HS-SPME方法不需要溶剂萃取和净化等繁琐操作,具有快速、灵敏、准确、环保等优点,为植物油中PAEs的灵敏筛检提供了新的方法和技术。

## Abstract (en)

Phthalate esters (PAEs) are used as additives to enhance the pliability and malleability of plastics. These substances frequently migrate from packaging materials to vegetable oils because of the absence of covalent bonds. Over time, this migration could result in the accumulation of PAEs in the human body through ingestion, contributing to various diseases. Therefore, accurate qualitative and quantitative analyses of PAEs in vegetable oils are imperative to assess the origins of contamination and investigate their toxicity, degradation, migration, and transformation patterns. However, the concentration of PAEs in most samples is low, and the composition of vegetable oils is complex. Thus, PAEs must be enriched and purified using appropriate sample pretreatment procedures before analysis. Common methods for pretreating PAEs in oil include solid-phase extraction (SPE), dispersive SPE, and magnetic SPE. These techniques require time-consuming and labor-intensive procedures such as oil dissolution, solvent extraction, and degreasing. These approaches also require numerous solvents and containers, increasing the risk of sample cross-contamination. Solid-phase microextraction (SPME) integrates sampling, extraction, purification, concentration, and injection into a single process, significantly accelerating analytical testing and reducing the potential for sample cross-contamination. In headspace (HS) mode, the analytes achieve equilibrium on the coating and are extracted in the gas phase. The fibers are shielded from nonvolatile and high-relative molecular mass substances in the sample matrix. Thus, SPME is an ideal method for extracting volatile compounds in vegetable oils. When HS-SPME coupled with gas chromatography-mass spectrometry (GC-MS), it can achieve the rapid screening of PAEs in vegetable oil.

In this study, an SPME with cyclodextrin-based hypercrosslinked polymers (BnCD-HCP) coated on stainless steel fibers was employed to extract PAEs from vegetable oil. The structure and morphology of the polymers were characterized using Fourier-transform infrared spectroscopy, nuclear magnetic spectroscopy, and scanning electron microscopy. BnCD-HCP exhibited high stability and diverse interactions, including *π-π*, hydrophobic, and host-guest interactions. The oil samples were incubated with methanol, and the PAEs were extracted from the headspace using the probe. The optimal extraction parameters included an extraction time of 20 min, extraction temperature of 50 ℃, desorption time of 4 min, and desorption temperature of 275 ℃. The BnCD-HCP/HS-SPME method was evaluated under optimized experimental conditions. The limits of detection (LODs) and quantification (LOQs) were determined by applying signal-to-noise ratios (*S/N*) of 3 and 10, respectively. Method accuracy was evaluated using relative standard deviations (RSDs). Single-needle precision was evaluated by conducting three consecutive analyses at 3 h intervals within a day. Inter-needle precision was assessed by conducting the same analyses (three replicates) with differently coated fibers. The 12 PAE compounds exhibited good linearity with correlation coefficients (*R*^2^) of at least 0.99. The LODs and LOQs ranged from 0.21 to 3.74 μg/kg and from 0.69 to 12.34 μg/kg, respectively. The RSDs were in the range of 1.8%-11.4% and 5.1%-13.9% for the single-needle and needle-to-needle methods, respectively. The proposed method was applied to soybean, peanut, and sunflower oils, and two PAEs were found in all three oils. Moreover, the method demonstrated good precision (RSD=1.17%-11.73%) and recoveries (72.49%-124.43%). Compared with other methods, the developed method was able to extract many target analytes and had a low or comparable LOD and high recovery. More importantly, this method does not require tedious operations such as solvent extraction and purification. Consequently, the developed method can be used to extract not only PAEs in oils but also other substances with a high lipid content.

邻苯二甲酸酯(PAEs)在塑料制造中广泛用作添加剂,以提升聚合物材料的柔韧性、延展性和耐久性^[1]^。由于PAEs和塑料之间没有共价键结合且具有亲脂性,PAEs容易从塑料包装材料迁移到油脂类食品中^[2]^,人类摄入后在体内积累,从而导致罹患先天性异常、肝肾损害、不孕症、内分泌紊乱和癌症等一系列疾病的风险^[3,4]^。因此,对油脂中的PAEs进行准确的定性和定量分析对于评估污染的来源以及研究其毒性、降解、迁移和转化方式至关重要。目前,气相色谱法(GC)是最常用的PAEs检测技术,然而,由于PAEs含量相对较低,与油脂同属于酯类化合物,且油脂基质成分复杂,在色谱分析之前必须采用合适的样品前处理操作对油脂样品中的PAEs进行富集和净化。

目前,油脂中PAEs样品前处理的常见方法包括固相萃取(SPE)^[5,6]^、液液萃取^[7]^、分散固相萃取(dSPE)^[8]^和磁性固相萃取(MSPE)^[9]^等,这些方法涉及油脂溶解、液液萃取和除脂等多步耗时耗力的操作,从而增加了样品交叉污染的风险^[8,9]^。固相微萃取(SPME)将采样、提取、纯化、浓缩和注射整合到一个流程中,不仅大大加快了分析检测的速度,且有效减少了样品交叉污染的可能性^[10,11]^。在顶空模式下,分析物在萃取涂层上平衡并在气相中提取,纤维免受样品基质中非挥发性和高相对分子质量物质的污染,非常适合分析油性基质中的挥发性化合物^[12]^。在SPME技术中,涂层的性能是提高萃取效率的关键,目前用于油脂中挥发物顶空萃取的SPME纤维包括聚二甲基硅氧烷(PDMS)及其复合物^[13]^、碳纳米管^[14]^、石墨烯^[15]^和金属有机骨架(MOFs)^[16]^等。然而,这些纤维在制备过程和性能等方面仍然存在一些缺点。例如,PDMS涂层缺乏多孔性、比表面积小导致富集因子低;碳基多孔吸附剂的识别作用单一,结构控制不够灵活;而MOFs容易受到湿气、溶剂、酸和碱等外界环境的干扰。因此,开发具有高选择性、高稳定性和低成本的涂层纤维用于油脂中PAEs的顶空萃取仍是一个挑战。

*β*-环糊精(*β*-CD)作为一种经济且可持续生产的大环主体化合物^[17,18]^,引起了众多研究人员的兴趣,*β*-CD用作样品前处理功能因子时能够与目标分析物之间形成“主客体相互作用”^[19,20]^,从而实现对PAEs的特异性识别,例如,Zhang等^[21]^开发了一种由*β*-CD改性的核壳型Au@Ag@*β*-CD,通过主客体识别将PAEs吸附到疏水腔中,达到富集的目的。然而,环糊精分子虽然具有识别客体分子的空腔,但本身并不具备孔隙结构,这可能导致其在吸附过程中的效率较低,因此需要将环糊精固定在多孔支撑材料上或将其制备为多孔材料。超交联聚合物(HCPs)具有比表面积高、制备简单、单体的选择范围宽、合成条件温和以及成本低等优点^[22]^,在样品前处理和污染物去除领域得到广泛应用^[23⇓-25]^。Zhou等^[26]^以富含芳基的*β*-CD-聚氨酯前体通过Friedel-Crafts反应合成了具有高比表面积和高效吸附双酚A的超交联*β*-环糊精聚氨酯材料;苄基化环糊精分别与对二氯苄^[27]^或甲醛缩二甲醇(FDA)^[28]^交联,得到的环糊精HCPs可用于吸附酚类化合物。从化学结构来看,环糊精超交联聚合物能够通过疏水、*π-π*和主客体相互作用等作用方式识别客体分子,有望应用于复杂基质中PAEs的萃取。

本研究制备了苄基化环糊精超交联聚合物(BnCD-HCP)涂覆不锈钢固相微萃取探针,并对BnCD-HCP及其探针的形貌、结构和热稳定性进行了表征。利用其芳香环和环糊精双功能结构单元与PAEs之间的疏水、*π-π*和主客体识别作用,采用顶空萃取模式实现了对植物油中PAEs的高效萃取和富集,通过优化影响顶空固相微萃取(HS-SPME)过程的吸附和解吸条件参数,结合气相色谱-质谱(GC-MS)联用技术,建立了植物油中PAEs的检测新方法。

## 1 实验部分

### 1.1 仪器与试剂

Agilent 8890 GC-5977B气相色谱-质谱联用仪(美国Agilent公司);自制固相微萃取手柄以及10 mL顶空样品瓶(美国Supelco公司); Nicolet傅里叶红外光谱仪(FT-IR,美国Thermo Scientific公司); AVANCE 400^13^C固体核磁(^13^C NMR,瑞士Bruker公司); JSM-7610F扫描电子显微镜(中国捷欧路公司); HS-TGA-101热重分析仪(TGA,上海和晟公司)。

PAEs标准品包括邻苯二甲酸二甲酯(DMP,纯度99.5%)、邻苯二甲酸二乙酯(DEP,纯度99.0%)、邻苯二甲酸二异丁酯(DIBP,纯度98.0%)、邻苯二甲酸二丁酯(DBP,纯度99.0%)、邻苯二甲酸二(4-甲基-2-戊基)酯(BMPP,纯度98.0%)、邻苯二甲酸二(2-乙氧基乙基)酯(DEEP,纯度98.0%)、邻苯二甲酸二戊酯(DPP,纯度99.0%)、邻苯二甲酸二己酯(DHXP,纯度98.0%)、邻苯二甲酸丁苄酯(BBP,纯度99.0%)、邻苯二甲酸二(2-正丁氧基乙基)酯(DBEP,纯度95.0%)、邻苯二甲酸二苯酯(DPhP,纯度98.0%)和邻苯二甲酸二正辛酯(DNOP,纯度98.0%)均购于阿拉丁化学有限公司(上海)。质量浓度为10 mg/L的15种PAEs混合标准储备液用甲醇配制,并在4 ℃冰箱内保存,后续按照实验要求以空白油样稀释成所需的系列PAEs标准工作溶液。

*β*-CD、溴化苄(BnBr,纯度98.0%)、氢化钠(NaH,纯度60.0%)、FDA(纯度98.0%)、氯化铁(FeCl_3_,纯度98.0%)和无水硫酸钠(Na_2_SO_4_,纯度99.0%)均购于百灵威科技有限公司(北京);二氯乙烷(DCE,纯度99.5%)、二氯甲烷(DCM,纯度99.5%)、甲醇(MeOH,纯度99.9%)、丙酮(纯度99.5%)、*N*,*N*-二甲基甲酰胺(DMF,纯度99.5%)、氨水(NH_3_·H_2_O,25.0%~28.0%)、石油醚和乙酸乙酯(EtOAc,纯度99.5%)均购于麦克林生化科技股份有限公司(上海)。SYLGARD^TM^184_聚二甲基硅氧烷高透明灌封胶购于道康宁公司(上海)。空白油样橄榄油由中检集团中原农食产品检测(河南)有限公司提供,花生油、大豆油和葵花油均购于郑州当地超市。

### 1.2 色谱和质谱分析条件

HP-5MS 5%Phe毛细管柱(30 m×250 μm×0.25 μm,美国Agilent公司);柱温程序设定:60 ℃保持1 min,以20 ℃/min的速率升温至220 ℃保持1 min;以5 ℃/min的速率升温至250 ℃保持1 min;再以20 ℃/min的速率升温至290 ℃保持3 min。采用不分流进样模式,超纯氦气作为载气,流速为1 ℃/min。离子源温度、进样口温度和四极杆温度分别为270、280和150 ℃。溶剂延迟时间设置为7.7 min,扫描模式采用选择性离子检测(SIM)模式。

12种PAEs的特征离子见表1。

**表1:** 12种PAEs的保留时间、定性和定量离子

Analyte	Abbreviation	Retention time/min	Qualitative ions (*m/z*)	Quantitative ion (*m/z*)
Dimethyl phthalate	DMP	7.730	163, 77, 194, 133	163
Diethyl phthalate	DEP	8.570	149, 177, 105, 222	149
Diisobutyl phthalate	DIBP	10.257	149, 223, 104, 167	149
Dibutyl phthalate	DBP	10.985	149, 223, 205, 104	149
Bis(4-methyl-2-pentyl) phthalate	BMPP	12.008	149, 167, 85, 251	149
Di(2-ethoxyethyl) phthalate	DEEP	12.376	72, 149, 104, 193	149
Dipentyl phthalate	DPP	12.722	149, 237, 219, 104	149
Dihexyl phthalate	DHXP	14.833	149, 251, 104, 23	149
Butyl benzyl phthalate	BBP	14.989	149, 91, 206, 104	149
Bis(2-*n*-butoxyethyl) phthalate	BBEP	16.433	149, 101, 85, 193	149
Diphenyl phthalate	DPhP	17.524	225, 77, 104, 153	225
Dioctyl phthalate	DNOP	20.739	149, 279, 104, 261	149

### 1.3 BnCD-HCP涂层纤维的制备

BnCD-HCP参考文献[28]合成,制作涂层前以甲醇进行24 h索氏提取以除去未反应的单体。将直径0.20 mm、长度20.0 cm的不锈钢丝的一端用浓盐酸腐蚀约30 min后用一级水、甲醇、丙酮进行超声浴清洗,室温干燥。将干燥后的不锈钢插入预先调配好的PDMS高透明灌封胶(主剂与硬化剂质量比为10∶1)中,迅速拔出,然后使用薄层玻璃片擦拭掉多余的PDMS高透明灌封胶。将不锈钢丝垂直插入含有BnCD-HCP粉末的离心管中,进行几次旋转,将BnCD-HCP沾到不锈钢丝表面,涂层长度2 cm,然后将涂层纤维置于120 ℃烘箱中30 min。对照实验使用仅涂敷PDMS的涂层纤维。

### 1.4 HS-SPME萃取过程

所有操作避免使用塑料容器以减少PAEs的污染,玻璃器皿使用二氯甲烷、丙酮和正己烷依次进行冲洗。萃取过程简要描述如下^[29]^:用顶空瓶称取(1.00±0.01) g食用植物油样品,同时添加1 mL甲醇作为基质改进剂,接着,将自制的BnCD-HCP纤维顶空固定在顶空样品瓶口,并在50 ℃下进行超声处理,持续20 min。萃取完成后,将BnCD-HCP涂层纤维暴露在进样口4.5 min对PAEs进行解吸。图1展示了BnCD-HCP的合成及其涂层纤维的制备和HS-SPME的操作流程。

**图1:**
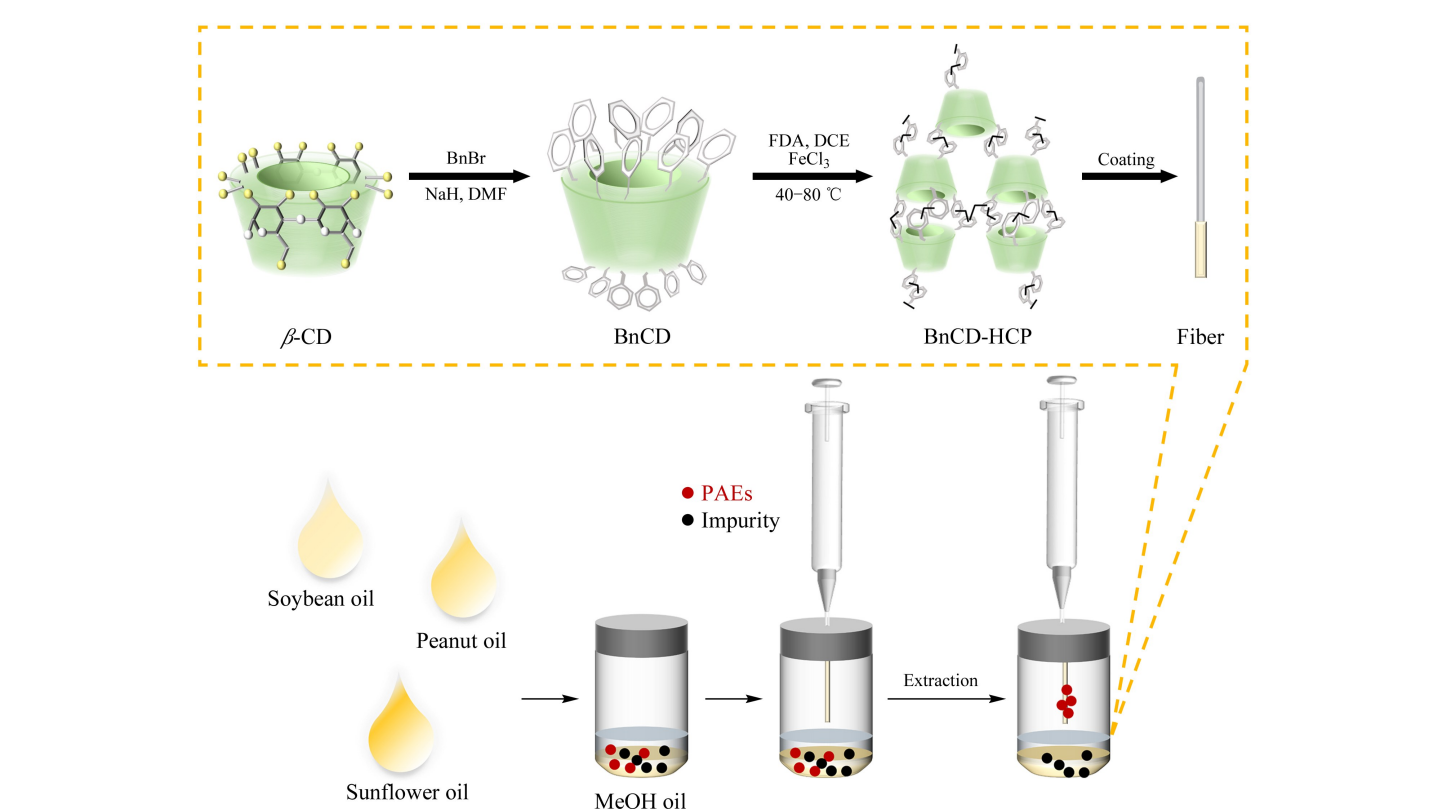
BnCD-HCP涂层纤维的制备流程及HS-SPME过程图

## 2 结果与讨论

### 2.1 BnCD-HCP材料及其涂层纤维的表征

采用FT-IR和^13^C NMR分析了材料的化学结构,结果如图2a、2b所示。通过与*β*-CD的红外谱图对比,可以观察到BnCD在1453 cm^-1^处出现苯环特征峰,且在3250 cm^-1^处*β*-CD上的羟基特征峰消失,表明*β*-CD已被完全苄基化。在BnCD-HCP红外谱图中,存在1453~1600 cm^-1^处的苯环特征峰、1023 cm^-1^处的醚键特征峰和2850 cm^-1^处的亚甲基特征峰,证明材料成功合成。同样,图2b的^13^C NMR谱图中,化学位移128处的峰归属于苯环碳原子,化学位移72和36处的峰分别归属于苄基碳和糖环上亚甲基碳原子,与文献[28]报道结果一致。

**图2:**
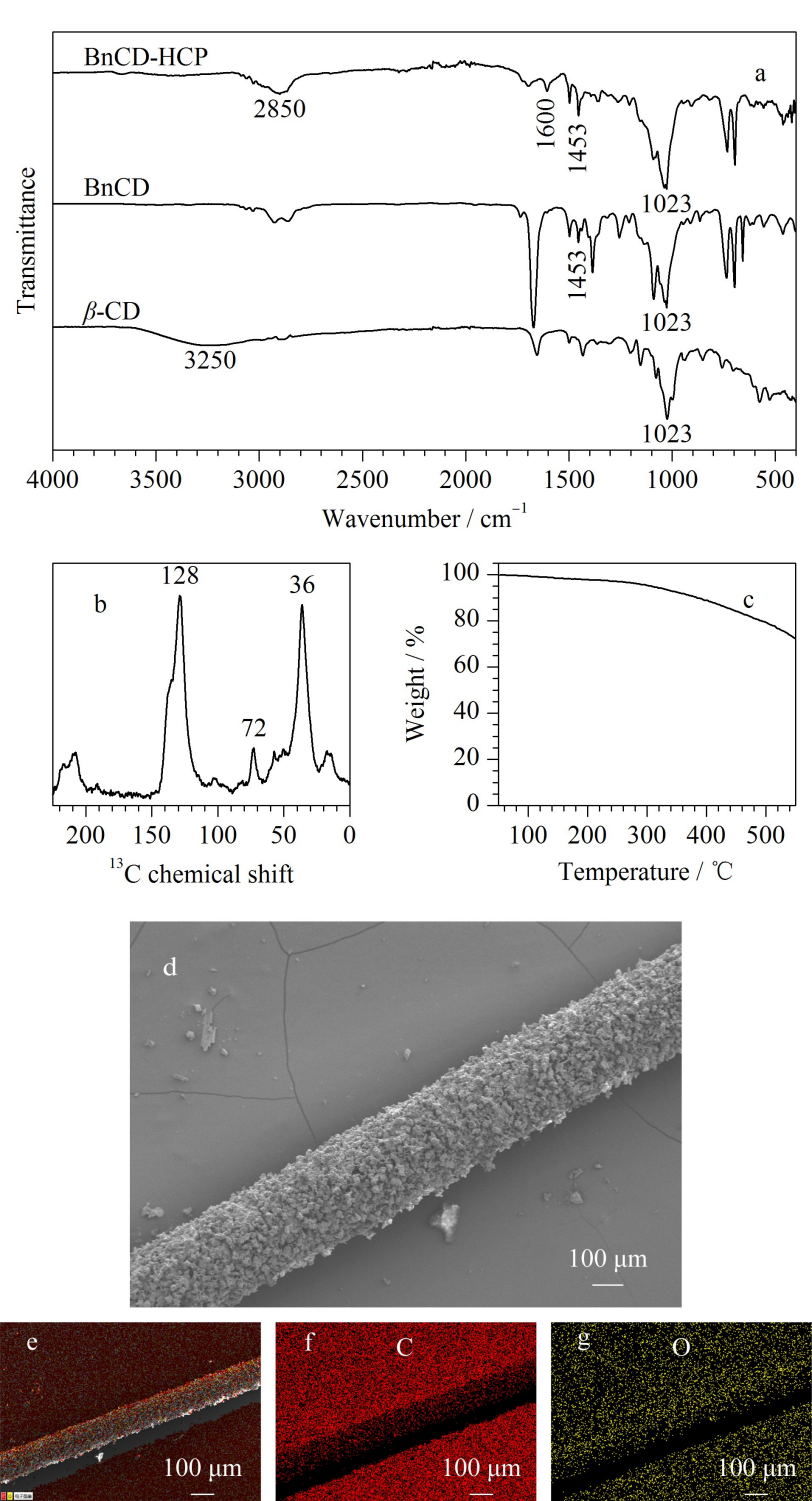
(a)BnCD-HCP、BnCD和*β*-CD的FT-IR光谱图, (b)BnCD-HCP的^13^C NMR图,(c)BnCD-HCP的热重分析图,(d)BnCD-HCP的扫描电镜图,(e~g)能谱分析(EDS)元素组成图

由于HS-SPME在进样口高温解吸释放目标物,因此材料的热稳定性至关重要。从图2c热重分析(TGA)可观察到BnCD-HCP在300 ℃时仍能保持95%重量,这表明BnCD-HCP具有出色的热稳定性。因此,可以确定BnCD-HCP能够满足在275 ℃解吸的要求,确保其在高温条件下的可靠应用。通过SEM对SPME涂层纤维的表面形貌进行了表征(图2d),可以观察到不锈钢丝表面包裹着一层均匀的BnCD-HCP涂层。能谱分析(EDS)元素组成分析表明C和O元素在涂层纤维中的分布呈现出致密而均匀的特征(图2e~g)。

### 2.2 HS-SPME萃取条件优化

为了优化BnCD-HCP包覆纤维对PAEs的萃取条件和参数,本研究以经过检验的玻璃瓶装橄榄油为空白样品,通过添加25 μg/kg的标准品,考察萃取时间、萃取温度、解吸时间和解吸温度等参数对BnCD-HCP萃取效果的影响。

首先,在10~30 min的范围内考察了萃取时间对PAEs萃取效率的影响(图3a)。除了相对分子质量较小的DMP和DEP的峰面积在萃取时间为10 min时达到最大值外,其他PAEs的峰面积均在20 min时达到最大值,之后峰面积逐渐减小,可能的原因是PAEs具有挥发性,可在BnCD-HCP涂层纤维上快速达到平衡,而随着萃取时间的延长,干扰物会与PAEs竞争吸附位点从而导致萃取效率下降。因此最优的萃取时间选为20 min。

**图3:**
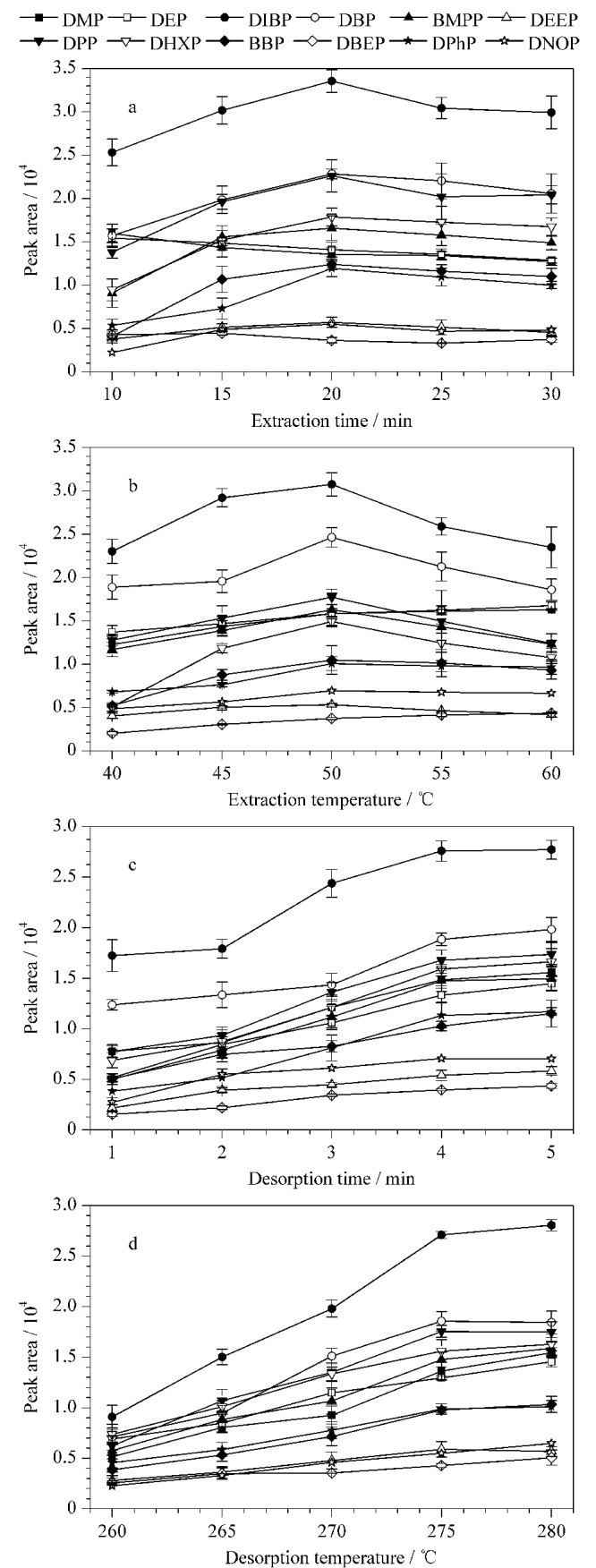
(a)萃取时间、(b)萃取温度、(c)解吸时间和(d)解吸 温度的优化(*n*=3)

萃取温度不仅影响萃取速率,而且影响涂层的吸附能力,因此萃取温度在顶空固相微萃取中起着至关重要的作用。在顶空模式下,微萃取包括两个过程:一是分析物从基质中释放,二是纤维涂层对分析物的吸附。提高温度会让分析物更有效地转移到样品的顶部空间,使得分析物可以更好地吸附到SPME纤维上。因此,在40~60 ℃内以5 ℃的间隔优化了温度对PAEs萃取效率的影响,从图3b可知,DMP和DEP等低相对分子质量PAEs的萃取效率在较低温度下达到最大,而高相对分子质量PAEs的萃取效率随着温度的增加,呈现先增加后减小的趋势。PAEs挥发性较强,导致吸附在纤维上的PAEs直接热解吸,所以过高的萃取温度不但会降低PAEs的萃取效率,而且不利于吸附。因此,接下来萃取实验在50 ℃下进行。

在解吸过程中,解吸时间和解吸温度是直接影响萃取效率的关键因素。为了达到最佳的萃取效率,在1~5 min范围内研究了解吸时间的影响(图3c)。当解吸时间从1 min增加到4 min时,峰面积先逐渐增加,然后保持不变,因此,最佳解吸时间为4 min。因为纤维表面涂层在高温条件下不可避免地会发生损失,导致BnCD-HCP纤维的萃取性能下降,所以在HS-SPME过程中选择一个合理的解吸温度至关重要。在低温条件下,纤维上吸附的分析物可能不能完全解吸,进而影响后续的实验结果。我们考察了解吸温度为260~280 ℃时对萃取效率的影响,由图4d可知,当解吸温度从260 ℃增至275 ℃时,色谱峰面积逐渐增大,当温度超过275 ℃时,峰面积基本不变,因此确定275 ℃为最佳解吸温度。

**图4:**
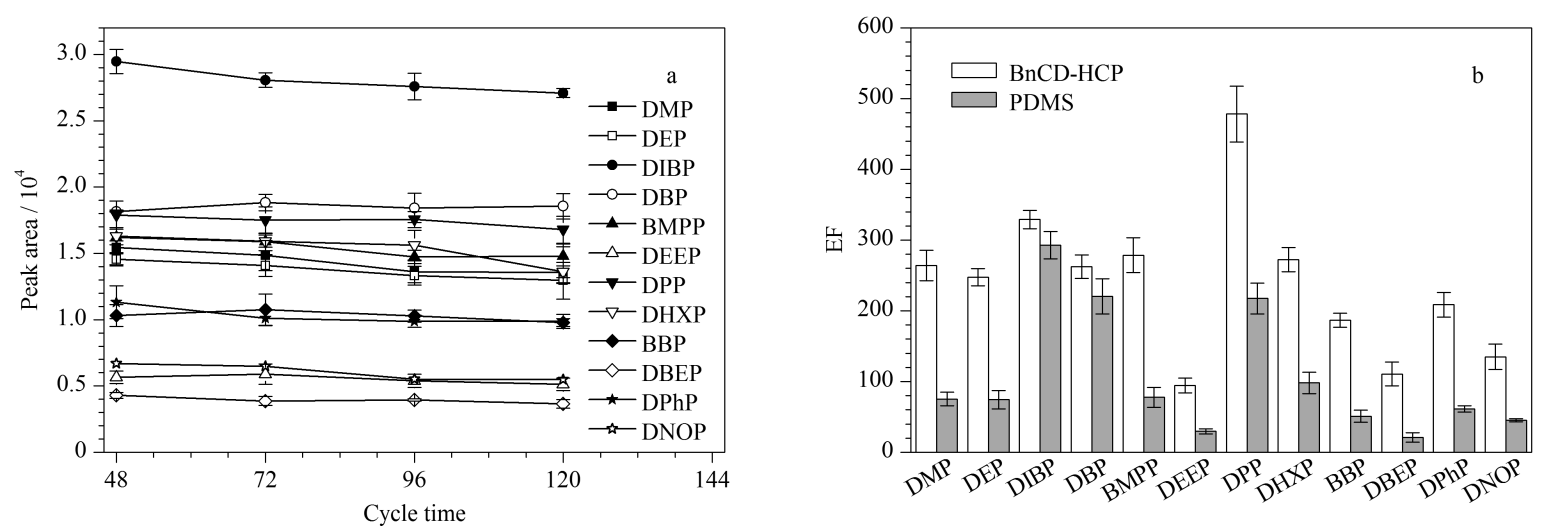
(a)BnCD-HCP涂层纤维使用次数对萃取效率的影响、(b)BnCD-HCP与自制PDMS涂层纤维的富集因子对比图(*n*=3)

### 2.3 BnCD-HCP使用寿命考察

经过超过120次的重复使用后,BnCD-HCP涂层纤维的萃取效果仍能保持在首次使用效果的80%以上(图4a),表明其具有卓越的耐久性和稳定性。为了深入研究BnCD-HCP涂层纤维在PAEs萃取中的优势,我们将其与PDMS涂层纤维进行了比较。从图4b中可以观察到,相较于未包覆的PDMS涂层纤维,BnCD-HCP固相微萃取纤维在所有PAEs的萃取中性能更好。

### 2.4 方法学验证

在最优萃取条件下,通过测量空白油样中掺入的12种分析物含量,绘制了PAEs的基质匹配标准曲线,标准溶液中PAEs的含量分别为0.5、1、3、5、10、25和50 μg/kg。以信噪比(*S/N*)=3和*S/N*=10分别计算了分析方法的检出限(LOD)和定量限(LOQ)。通过在空白橄榄油中添加25 μg/kg的PAEs来评估该方法单针重复性(一天内以3 h为间隔进行3次连续分析)和针间重复性(不同的涂层纤维进行3次相同的分析),以相对标准偏差(RSD)表示。由表2可知,12种PAEs在各自的线性范围内具有良好的线性关系(相关系数(*R*^2^)>0.99), LOD和LOQ分别为0.21~3.74 μg/kg和0.69~12.34 μg/kg,单针和针间的RSD为1.8%~11.4%和5.1%~13.9%。

**表2:** 12种PAEs的线性范围、相关系数、回归方程、检出限、定量限和重复性

Analyte	Linear range/ (μg/kg)	*R*^2^	Regression equation	LOD/ (μg/kg)	LOQ/ (μg/kg)	RSDs/% (*n*=3)	
						One fiber	Fiber to fiber
DMF	1.62-50	0.9998	*Y*=575.0*X*-173.9	0.49	1.62	6.9	12.9
DEP	1.65-50	0.9993	*Y*=551.1*X*-159.8	0.50	1.65	6.6	7.8
DIBP	0.69-50	0.9996	*Y*=1234.1*X*-268.2	0.21	0.69	3.6	6.5
DBP	0.73-50	0.9996	*Y*=749.7*X*+4.2	0.22	0.73	4.9	5.3
BMPP	1.68-50	0.9983	*Y*=578.2*X*+8.2	0.51	1.68	6.9	8.5
DEEP	10.69-50	0.9994	*Y*=222.0*X*+21.5	3.24	10.69	6.7	11.2
DPP	1.09-50	0.9968	*Y*=697.7*X*+267.9	0.33	1.09	6.5	5.1
DHXP	1.19-50	0.9977	*Y*=670.1*X*+220.4	0.36	1.19	7.6	7.1
BBP	2.31-50	0.9993	*Y*=379.7*X*+115.2	0.70	2.31	6.3	10.7
DBEP	2.48-50	0.9995	*Y*=186.2*X*-28.8	0.75	2.48	8.2	13.9
DPhP	1.82-50	0.9984	*Y*=474.0*X*-237.2	0.55	1.82	11.4	6.1
DNOP	12.34-50	0.9991	*Y*=224.1*X*-56.8	3.74	12.34	1.8	7.8

*Y*: peak area; *X*: content, μg/kg.

### 2.5 实际样品检测

将本方法用于分析大豆油、花生油和葵花油样品中12种PAEs的含量,成功检测到大豆油样品中含有DIBP(0.27 μg/kg)和DBP(0.25 μg/kg),花生油样品中含有DIBP(0.33 μg/kg)和DBP(0.30 μg/kg),葵花油样品中含有DIBP(0.21 μg/kg)和DBP(0.19 μg/kg)。为了评估该方法的准确性,在3个加标水平(1、5和25 μg/kg)下进行了加标回收试验, 12种PAEs在大豆油、花生油和葵花油中的回收率分别为72.49%~112.98%(RSD为1.27%~11.14%)、78.73%~114.41%(RSD为2.27%~11.73%)和77.45%~124.43%(RSD为1.17%~10.45%),具体结果见表3,相关谱图见图5。

**表3:** 12种PAEs在3个油样中3个水平下的加标回收率及精密度(*n*=3)

Analyte		Spiked/ (μg/kg)		Soybean oil								Peanut oil								Sunflower oil			
				Found/(μg/kg)		Recovery/%		RSD/%				Found/(μg/kg)		Recovery/%		RSD/%				Found/(μg/kg)		Recovery/%	RSD/%
DMP		0		ND		-		-				ND		-		-				ND		-	-
		1		1.00		99.79		5.26				0.99		99.22		2.56				0.79		79.22	1.49
		5		5.28		105.61		4.19				4.87		97.37		6.95				4.98		99.61	5.40
		25		23.01		99.02		5.31				26.25		105.01		9.22				25.29		101.18	1.17
DEP		0		ND		-		-				ND		-		-				ND		-	-
		1		0.95		94.81		4.89				1.05		104.61		6.98				1.01		101.04	4.29
		5		4.83		96.54		3.86				5.42		108.38		4.57				4.91		98.17	10.54
		25		24.72		98.87		5.19				27.95		111.78		7.77				26.59		106.38	4.68
DIBP		0		0.27		-		-				0.33		-		-				0.21		-	-
		1		1.20		92.55		6.82				1.47		114.41		5.13				1.34		113.05	6.19
		5		5.36		101.84		1.60				5.69		107.12		9.25				4.75		90.71	7.73
		25		26.56		105.14		2.76				23.93		96.07		5.13				24.19		95.93	8.44
DBP		0		0.25		-		-				0.30		-		-				0.19		-	-
		1		1.29		103.95		5.63				1.25		94.67		8.31				1.19		99.80	4.72
		5		4.69		88.89		7.87				5.22		98.33		6.94				4.89		93.94	4.52
		25		21.08		83.34		7.62				23.93		94.50		7.00				23.97		95.14	3.61
BMPP		0		ND		-		-				ND		-		-				ND		-	-
		1		0.97		97.18		7.36				1.08		107.86		4.24				1.13		112.99	4.62
		5		4.50		89.99		9.32				5.44		108.72		6.48				5.26		105.22	3.29
		25		25.30		101.21		8.67				26.12		104.48		6.49				22.15		88.59	8.35
DEEP		0		ND		-		-				ND		-		-				ND		-	-
		1		0.72		72.49		8.58				1.06		106.15		11.73				1.24		124.43	3.05
		5		4.78		95.69		8.42				4.63		92.64		4.72				5.40		108.03	1.50
		25		19.77		79.06		4.70				23.08		92.33		7.97				25.19		100.77	6.78
DPP		0		ND		-		-				ND		-		-				ND		-	-
		1		1.03		102.70		5.82				1.01		101.34		6.58				0.90		89.90	3.98
		5		5.40		107.96		4.51				4.36		87.22		11.68				4.78		95.65	10.11
		25		21.99		87.96		1.88				19.68		78.73		2.27				23.41		93.63	6.82
DHXP		0		ND		-		-				ND		-		-				ND		-	-
		1		0.99		99.44		6.22				0.92		91.79		6.36				0.97		96.77	7.64
		5		4.48		89.54		7.73				5.30		106.07		3.53				5.48		109.61	7.50
		25		23.78		95.13		3.67				22.22		88.88		8.77				21.76		87.05	4.94
BBP		0		ND		-		-				ND		-		-				ND		-	-
		1		1.07		107.48		5.62				1.08		107.94		7.60				0.87		86.69	2.08
		5		5.43		108.59		10.28				5.21		104.24		5.11				5.13		102.64	8.50
		25		24.27		97.07		4.87				26.85		107.40		9.43				25.50		101.99	9.75
DBEP		0		ND		-		-				ND		-		-				ND		-	-
		1		1.05		104.80		7.21				1.02		101.90		8.59				1.10		110.02	10.03
		5		5.01		100.24		5.97				5.36		107.17		10.97				4.79		95.87	7.34
		25		28.25		112.98		6.62				26.15		104.59		8.73				28.29		113.15	8.52
DPhP		0		ND		-		-				ND		-		-				ND		-	-
		1		1.02		101.65		10.26				1.00		99.89		5.96				1.04		103.73	7.89
		5		5.26		105.12		1.27				4.83		96.64		2.72				4.72		94.39	6.46
		25		27.16		108.65		8.41				26.24		104.97		6.82				25.07		95.00	4.58
DNOP		0		ND		-		-				ND		-		-				ND		-	-
		1		0.88		83.50		6.97				1.06		105.95		7.33				0.92		99.25	6.43
		5		4.37		101.91		11.14				5.49		109.73		9.29				3.87		77.45	8.71
		25		26.16		106.90		9.68				27.46		109.84		9.48				22.38		110.19	7.37

ND: not detected.

**图5:**
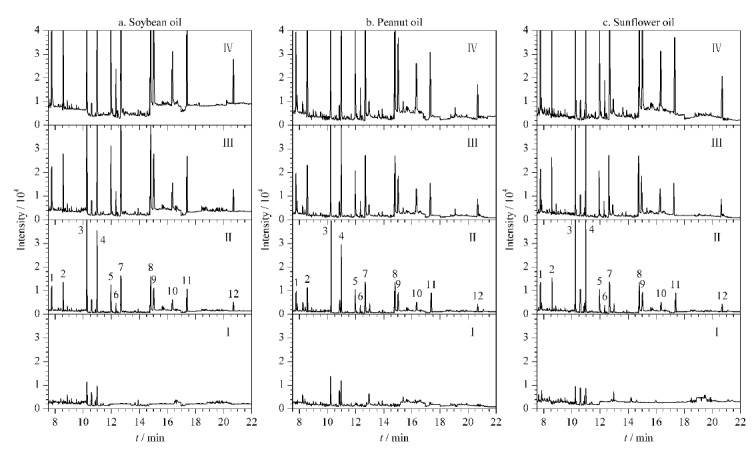
(a)大豆油、(b)花生油和(c)葵花籽油经过HS-SMPE处理后目标PAEs的总离子流图

### 2.6 与其他方法比较

将本文方法与其他方法进行了对比,结果如表4所示,与MSPE和dSPE相比,本方法采用的HS-SPME技术减少了溶剂消耗和样品间的交叉污染,避免了浓缩、净化、氮吹等一系列会导致误差的操作,所萃取的目标分析物数量多,与文献[5⇓-7,30⇓⇓-33]相比有更低或相当的检出限,回收率及RSD符合要求。该法除了可用于油中PAEs的检测外,还有望用于巧克力、油脂薯片、方便面等其他脂含量高的食品。

**表4:** 本方法与其他方法的比较

Coating	Methods	Number of PAEs	Samples	LOD/ (μg/L)	LOQ/ (μg/L)	Recovery/ %	RSD/ %	Ref.
SWCNTs	SPE/GC-MS	6	camellia oil	-	10.0-30.0^a^	86.4-111.7	5.4-10.4	[5]
DSMNPs	dSPE/HPLC/UV	5	water	0.5-5.0	3.0-10.0	85.7-105.5	3.9-5.7	[6]
Co-MNPC@MIPs	MSPE/GC-FID	5	edible oil	10.0-25.0	32.0-69.0	81.6-102.2	3.3-12.0	[7]
G/PVC	HS-SPME/GC-FID	4	vegetable oil	60.0-80.0	200.0-300.0	87.0-112.0	8.1-10.5	[30]
MWCNTs	dSPE/GC-MS	7	virgin olive oil	6.0-50.0	-	87.0-111.0	3.7-7.6	[31]
PDMS	DI-SPME/GC-QqQ MS	9	vegetable oil	15.0-144.0^a^	25.0-523.0^a^	-	1.3-11.8	[32]
PDMS/DVB	HS-SPME/GC-MS	4	vegetable oil/soft drink	0.1-3.3	0.2-11.1	84.5-102.1	1.6-4.9	[33]
BnCD-HCP	HS-SPME/GC-MS	12	vegetable oil	0.21-3.74^a^	0.7-12.3^a^	72.5-124.4	1.2-11.7	this work

SWCNTs: single-walled carbon nanotubes; DSMNPs: diatomaceous earth-supported magnetite nanoparticles; Co-MNPC@MIPs: cobalt magnetic nanoporous carbon molecularly imprinted polymers; G/PVC: graphene/polyvinylchloride; MWCNTs: multi-walled carbon nanotubes; DVB: divinylbenzene; dSPE: dispersive solid-phase extraction; DI-SPME: direct immersion solid-phase microextraction extraction; a: the unit is μg/kg.

## 3 结论

本研究成功制备了一种具有良好稳定性和高重复性的HS-SPME涂层纤维,该涂层表现出良好的萃取性能。将其与GC-MS结合并对萃取和解吸条件进行优化,建立了一种具有较低检出限、较高重复性和回收率的分析方法。方法有效避免了溶剂提取、净化、氮吹复溶等繁琐操作所导致的误差,具有绿色、快速、灵敏、准确等优点,为植物油中PAEs的检测提供了新的方法和技术,同时进一步拓展了环糊精大环主体分子和HCPs的应用范围。
